# The Wilms Tumor-1 (WT1) rs16754 polymorphism is a prognostic factor in acute myeloid leukemia (AML): a meta-analysis

**DOI:** 10.18632/oncotarget.8117

**Published:** 2016-03-16

**Authors:** Jianting Long, Shi Fang, Qiangsheng Dai, Xiaolian Liu, Wanshou Zhu, Shenming Wang

**Affiliations:** ^1^ Department of Medicinal Oncology, The First Affiliated Hospital, SUN Yat-Sen University, Guangzhou 510080, China; ^2^ Department of Clinic Nutrition, The First Affiliated Hospital, SUN Yat-Sen University, Guangzhou 510080, China; ^3^ Department of Hematology, Gaozhou People's Hospital, Gaozhou 525200, China; ^4^ Department of Vascular Surgery, The First Affiliated Hospital, SUN Yat-Sen University, Guangzhou 510080, China

**Keywords:** acute myeloid leukemia, Wilms tumor gene 1, association, meta-analysis

## Abstract

Although a number of studies suggested that WT1 rs16754 polymorphism might be related to decreased relapse free survival (RFS) and overall survival (OS). The results remain controversial. Published reports were searched in PubMed, EMBASE, and Google Scholar. Twelve publications with 3903 patients had met the inclusion criteria and were subjected to further examination. We found WT1 rs16754 polymorphism was significantly associated with OS in AML (OR = 0.62; 95% CI 0.52 − 0.75; *p* < 0.00001; I^2^ = 47%). WT1 rs16754 polymorphism was also significantly associated with RFS in AML (OR = 0.69; 95% CI 0.57 − 0.83; *p* < 0.001; I^2^ = 46%). In the subgroup analyses of age, race, and subtype of AML, WT1 rs16754 polymorphism was a independent favorable-risk marker. In conclusion, WT1 rs16754 polymorphism is associated with better survival of AML. It could be used as a cost-effective prognostic biomarker for AML.

## INTRODUCTION

Acute myeloid leukemia (AML) is a serious disease of the hematopoietic system characterized by de-differentiation and uncontrolled proliferation of immature hematopoietic precursor cells in the bone marrow [1]. It is usually based on many parameters including age, white blood cell count, cytogenetic abnormalities, and specific mutations [2]. AML results from the accumulation of genetic and epigenetic alterations during the multistep process of tumorigenesis, including activation of oncogenes and/or inactivation of tumor suppressor genes.

The Wilms’ tumor gene 1 (WT1), located at chromosome 11p13, was firstly cloned in 1990 as a suppressor in Wilms’ tumor [3]. WT1 regulates transcription, RNA metabolism, translation and both oncogenic and tumor suppressor functions [4]. Since the expression of WT1 in many solid cancers is upregulated, it is considered as a potential oncogene [5]. Although the pathogenesis of WT1 in leukemia has not been completely revealed, the phenomenon that low expression of WT1 and high expression of WT1 are accompanied with clinical remission and relapse respectively shows that WT1 may be a potential prognostic factor in acute leukemia [6].

Although a number of studies suggested that WT1 rs16754 polymorphism might be related to decreased relapse free survival (RFS) and overall survival (OS). The results remain controversial [7-18]. In this study, meta-analysis of the association between WT1 rs16754 polymorphism and OS and RFS of AML was conducted. Twelve studies involving 3903 patients with AML are pooled in the meta-analysis.

## RESULTS

### Characteristics of the studies

**Figure 1:**
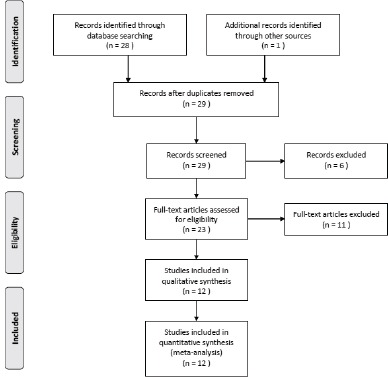
Study flow diagram of included studies

The flow chart in Figure 1 summarizes this literature review process. Twelve publications with 3903 AML patients had met the inclusion criteria and were included in this meta-analysis. Seven studies were conducted in Caucasians, 3 were conducted in Asians, and the remaining 2 studies were conducted in mixed ethnic groups. Two studies included child and the remaining studies included adults. Characteristics of the included studies are shown in Table 1.

**Table 1:** Characteristics of the included studies

No.	First author	Year	Study design	Race	Age	Female (%)	Subtype	Sample size	Adjusted for OS	Adjusted for RFS
1	Damm	2010	Cohort	Caucasian	45	48	CN-AML	249	NPM1/FLT3 mutation status, age, Platelet, CEBPA mutation status, WBC	NPM1/FLT3 mutation status, Platelet, CEBPA mutation status, WBC
2	Hollink	2010	Cohort	Caucasian	9	40	Mix	272	NA	NA
3	Wagner	2010	Cohort	Caucasian	49	83	CN-AML	275	Age, IDH1 SNP rs11554137, NPM1/FLT3 mutation status, Platelets	NPM1 mutation status, CEBPA mutation status
4	Becker	2011	Cohort	Mix	62	50	CN-AML	433	NA	NA
5	Ho	2011	Cohort	Mix	10	45	NA	790	Risk group, WBC, race	Risk group, WBC, race
6	Renneville	2011	Cohort	Caucasian	51	46	Mix	511	NA	NA
7	Damm	2012	Cohort	Caucasian	47	48	CN-AML	269	ID1, age, BAALC expression, Platelets, FLT3-ITD, CEBPA mutation	ID1, Platelets, NPM1 mutation, CEBPA mutation
8	Choi	2012	Cohort	Asian	42	63	CN-AML	73	NA	NA
9	Luna	2012	Cohort	Caucasian	62	43	de novo AML	175	NPM1, FLT3-ITD, and CEBPA, expression levels of WT1, age, WBC count, platelet count, hemoglobin level, percentage of blood blasts, cytogenetic risk group, and FAB classification	NPM1, FLT3-ITD, and CEBPA, expression levels of WT1, age, WBC count, platelet count, hemoglobin level, percentage of blood blasts, cytogenetic risk group, and FAB classification
10	Luo	2014	Cohort	Asian	45	58	de novo non-M3 AML	182	Age, percentage of blood blasts, WT1 expression, WBC, FLT3	Age, percentage of blood blasts, WT1 expression, WBC, FLT3
11	Niavarani	2015	Cohort	Caucasian	45	55	CN-AML	469	Age, sex, WBC, secondary disease, performance status, FLT3-ITD, and NPM1 mutations	Age, sex, WBC, secondary disease, performance status, FLT3-ITD, and NPM1 mutations
12	Zhang	2015	Cohort	Asian	40	46	Mix	205	Risk stratification, allogeneic hematopoietic stem cell transplantation	Risk stratification, allogeneic hematopoietic stem cell transplantation

### Association between WT1 rs16754 polymorphism and OS of AML

**Figure 2:**
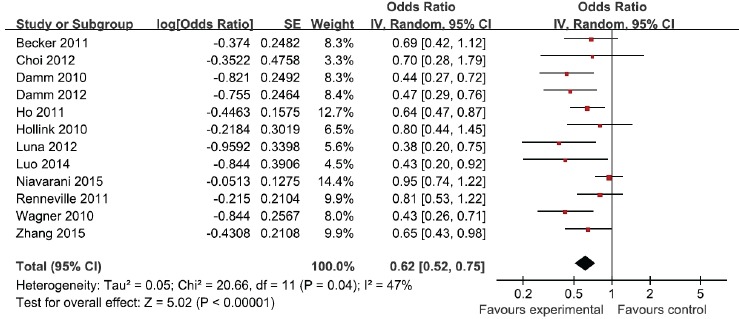
Meta-analysis of the association between WT1 rs16754 polymorphism and OS of AML

When all eligible studies were pooled into one dataset for the meta-analysis, we found WT1 rs16754 polymorphism was significantly associated with OS in AML (OR = 0.62; 95% CI 0.52 − 0.75; *p* < 0.00001; Figure 2). In the subgroup analysis of race, both Asians and Caucasians showed better OS in AML (OR = 0.61; 95% CI 0.43 − 0.85; *p* = 0.004; and OR = 0.60; 95% CI 0.44 − 0.81; *p* = 0.001). In addition, both de novo AML and CN-AML showed better OS in AML (OR = 0.40; 95% CI 0.24 − 0.67; *p* = 0.0004; and OR = 0.60; 95% CI 0.43 − 0.84; *p* = 0.001). In a further stratified analysis by age, both children and adults showed better OS in AML (OR = 0.67; 95% CI 0.51 − 0.88; *p* = 0.004; and OR = 0.60; 95% CI 0.47 − 0.75; *p* < 0.0001). The results were showed in Table 2.

**Table 2:** Results of the meta-analysis

	OR (95% CI)	*P* Value	*I*^2^ (%)	*p* Value
OS	0.62 (0.52-0.75)	<0.00001	47	0.04
Subtype				
de novo AML	0.40 (0.24-0.67)	0.0004	0	0.82
CN-AML	0.60 (0.43-0.84)	0.003	67	0.009
Race				
Asian	0.61 (0.43-0.85)	0.004	0	0.61
Caucasian	0.60 (0.44-0.81)	0.001	69	0.004
Age				
<18 years	0.67 (0.51-0.88)	0.004	0	0.50
>18 years	0.60 (0.47-0.75)	<0.0001	55	0.02
RFS	0.69 (0.57-0.83)	<0.001	46	0.04
Subtype				
de novo AML	0.42 (0.26-0.68)	0.0004	0	0.49
CN-AML	0.74 (0.62-0.88)	0.007	36	0.12
Race				
Asian	0.61 (0.42-0.88)	0.008	0	0.65
Caucasian	0.64 (0.49-0.85)	0.002	63	0.001
Age				
<18 years	0.74 (0.55-1.00)	0.05	0	0.50
>18 years	0.68 (0.54-0.85)	0.0006	55	0.02

### Association between WT1 rs16754 polymorphism and RFS of AML

**Figure 3:**
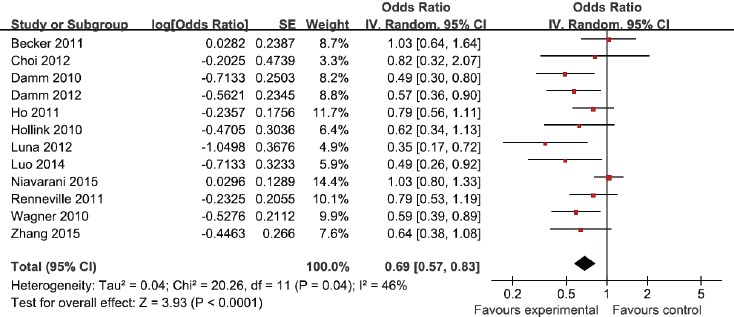
Meta-analysis of the association between WT1 rs16754 polymorphism and RFS of AML

As for RFS of AML, WT1 rs16754 polymorphism was found to be significantly associated with RFS (OR = 0.69; 95% CI 0.57 − 0.83; *p* < 0.001; Figure 3). In the subgroup analysis of race, both Asians and Caucasians with this polymorphism exhibited better RFS in AML (OR = 0.61; 95% CI 0.42 − 0.88; *p* = 0.008; and OR = 0.64; 95% CI 0.49 − 0.85; *p* = 0.002). In terms of AML subtype, both de novo AML and CN-AML displayed better RFS in AML (OR = 0.42; 95% CI 0.26 − 0.68; *p* = 0.0004; and OR = 0.74; 95% CI 0.62 − 0.88; *p* = 0.007). In the stratified analysis by age, a significantly better RFS was found among children (OR = 0.74; 95% CI 0.55 − 1.00; *p* = 0.05), and was also found among adults (OR = OR = 0.68; 95% CI 0.54 − 0.85; *p* = 0.0006). The results were listed in Table 2.

### Sensitivity analyses and publication bias

**Figure 4:**
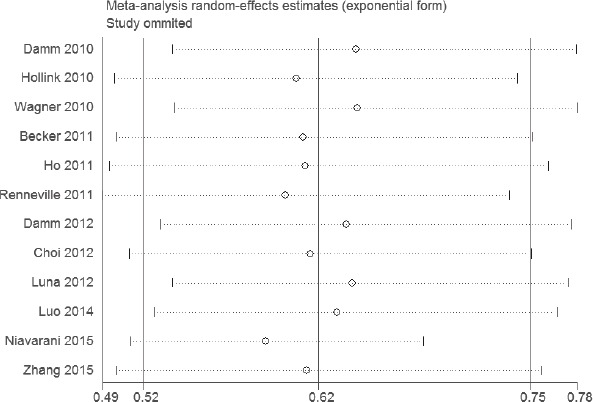
Sensitivity analysis of the association between WT1 rs16754 polymorphism and OS of AML

**Figure 5:**
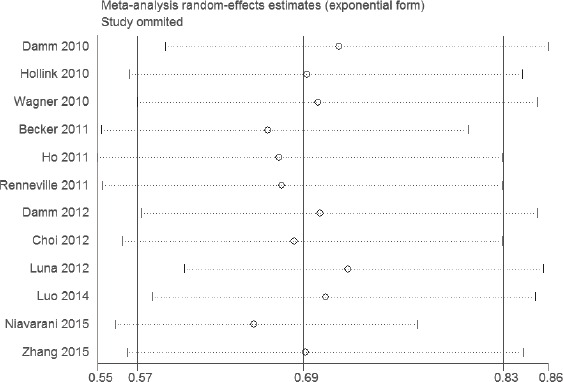
Sensitivity analysis of the association between WT1 rs16754 polymorphism and RFS of AML

**Figure 6:**
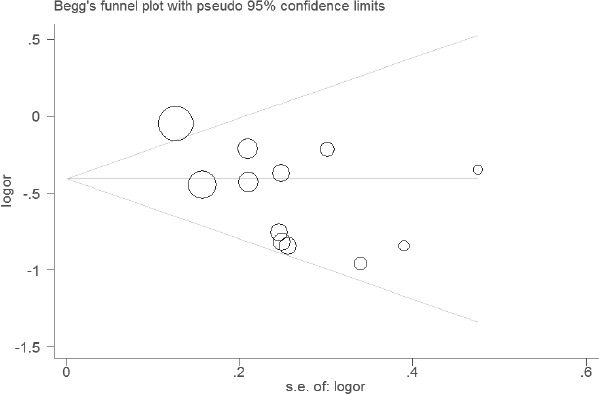
Funnel plot of the association between WT1 rs16754 polymorphism and OS of AML

**Figure 7:**
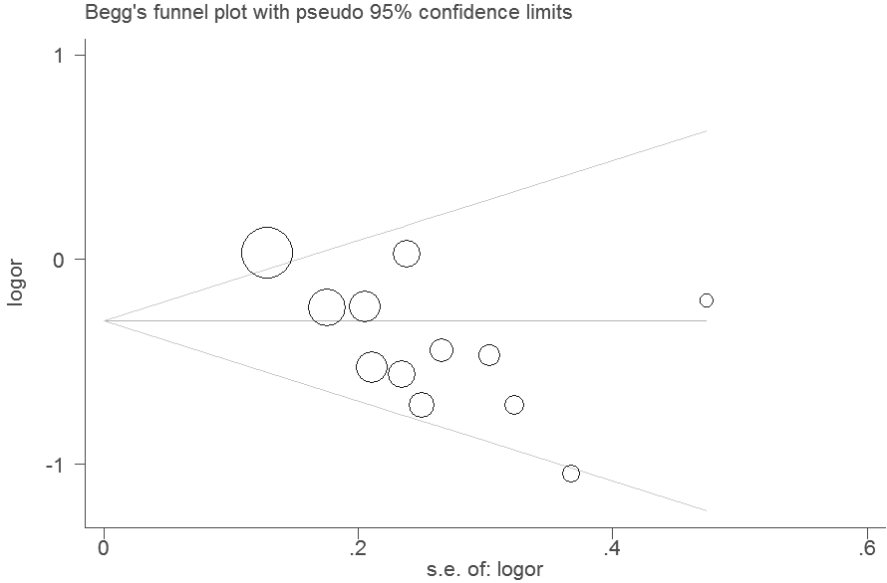
Funnel plot of the association between WT1 rs16754 polymorphism and RFS of AML

The results of sensitivity analyses showed that the estimates before and after the deletion of each study were similar (Figures 4 and 5). The shapes of the funnel plot seemed symmetrical (Figures 6 and 7), suggesting that there was no obvious publication bias.

## DISCUSSION

Many studies have investigated the relation of WT1 rs16754 polymorphism with prognosis of AML, but the conclusions are still controversial. In view of this issue, we comprehensively analyzed the association between WT1 rs16754 polymorphism and OS or RFS of AML via the method of meta-analysis, and found that WT1 rs16754 polymorphism affected the survival of AML. Thus, WT1 rs16754 polymorphism might be a prognostic factor of AML.

WT1 expression is detectable in CD34+ progenitor cells, but is down-regulated during the process of hematopoietic differentiation, and undetectable in mature leukocytes [19]. WT1 overexpression could be observed in the majority of AML patients at diagnosis, which disappeared when complete remission (CR) was achieved by chemotherapy. Hence, WT1 expression has been regarded as a potential biomarker for the detection of minimal residual dis-ease (MRD) in clinic [20]. Ujj et al. also suggested that disappearance of WT1-positivity during chemotherapy had a favorable effect on survival [21]. Casalegno-Garduño et al. indicated that WT1 is a suitable marker for the detection of minimal residual disease after SCT or chemotherapy [22]. Recently, analysis of WT1 expression in circulating RNA in plasma in AML patients could be a simple, convenient and noninvasive method to predict latent information about relapse [23]. WT1 rs16754 polymorphism consists in a change ofthe nucleotide adenine (A) into guanine (G). Zhang et al. found that the WT1 GG patients showed significantly higher WT1 mRNA expression than the WT1 GA/AA patients [18]. Thus, it might be the reason for why WT1 rs16754 polymorphism could influence the survival of AML.

We performed sensitivity analysis by excluding studies to verify the stability of results. However, this present study also has some limitations. First, the sample size was relatively small in subgroup analyses. Second, significant heterogeneity was detected in included studies and the accuracy of results would be affected in spite of utilizing the random-effects model to calculate pooled ORs. Third, we didn't explore gene-gene and gene-environment interactions because of the insufficient data. Finally, this study is a meta-analysis of case-control study and cohort study. Confounding cannot be avoided and should be considered.

In conclusion, WT1 rs16754 polymorphism is associated with better survival of AML. It could be used as a cost-effective prognostic biomarker for AML.

## MATERIALS AND METHODS

### Publication search

Published reports were searched in PubMed, EMBASE, and Google Scholar, with the following key words: “acute myeloid leukemia” and “Wilms’ tumor gene 1”. The MeSH terms, such as “leukemia, myeloid, acute” and “genes, wilms tumor”, were used. Publication language was not restricted in this search. Reference lists of articles retained for review were examined manually to further identify potentially relevant reports.

### Study selection

Two reviewers independently screened the abstracts of papers identified by the literature search, retrieved potentially relevant studies and determined study eligibility. Studies were included if: (1) study design was prospective or retrospective cohort study; (2) the exposure of interest was WT1 rs16754 polymorphism; (3) the studied reported relative risks (RRs), hazard ratios (HRs), or odds ratios (ORs) with corresponding 95% confidence intervals (CIs); and (4) the outcome was RFS or OS. If the same cohort was used in more than one publication, we included the publication that reported the results in greater detail or, if similar, the one with the largest number of cases. Data published only in abstract form were excluded. Case reports, review articles and commentary articles were also excluded.

### Data extraction

The following data including: the first author, year, study design, ethnicity of the study population, age, sex, subtype, number of patients included in analysis, adjusted factors, and OR with its 95% CI for OS and RFS were extracted from each eligible study by two investigators.

### Statistical analysis

The strength of association between WT1 rs16754 polymorphism and RFS and OS of AML was evaluated by calculating the OR and 95% CI. To assess the significance of OR, we conducted the Z test and we regarded it as significant difference when P value less than 0.05 was detected. Moreover, X2 based Q and I2 test were performed to evaluate the between-study heterogeneity and P<0.1 was defined as statistical significance. The random-effects was used to calculated the OR if significant heterogeneity existed. Otherwise, the fixed-effects model was applied. Furthermore, we also conducted subgroup analyses stratified by ethnicity (Caucasian and Asian), age (child and adult), and subtype of AML. Publication bias was assessed by asymmetry of funnel plots. In sensitivity analysis, we sequentially excluded each study on the software to evaluate the stability of the results. We conducted all the analyses by using software Review Manager 5.1 (Nordic Cochrane Center, Copenhagen, Denmark) and STATA 11.0 software (Stata Corporation, College Station, TX, USA).
